# Supplementary material for: Impact of a teacher-led school handwashing program on children’s handwashing with soap at school and home in Bihar, India
Source: PLoS One. 2020 Feb 27;15(2):e0229655. doi: 10.1371/journal.pone.0229655 (PMC7046199; doi:10.1371/journal.pone.0229655)
Supplement: Attachment — Submitted filename: PlosOne Comments_2019.docx [file pone.0229655.s001.docx]

PlosOne Comments:

Dear Authors, I appreciate the efforts of the authors in choosing the subject of great importance in today’s context for India.

However, I have reservations for its publications. The reasons for the same is enumerated below:

1. I am convinced with the background on the need of the study. However, there is limited explanation of “school of five programme”- the type, format, frequency, duration, modules used for training and whether the training model was piloted in any other areas before its implementation as an intervention. If yes, what were the challenges and operational issues faced by the investigators during the pilot and its validation. It is also not clear whether the focus of training programme was to have the behavioral changes among the students or mothers or both.
2. The research question is not clear as it states that they are reporting the evaluation done for the programme and at the same time it is said they want to measure the impact of the programme. Probably, they are interested in comparing the differences between the intervention and non-intervention arm. The measure of outcome is missing in the research question.
3. It is less evident whether there could be any behavior change in the context of this settings whether it is appropriate to have an impact study with one month of implementation.
4. The sampling of the villages and the schools explained in the methods are incomplete. The sampling frame, the sampling technique, the sample size calculation, the assumptions made to extrapolate the findings to the general population are inadequately explained.
5. The information on the methods of measuring the outcomes at school and the mother at the home settings are not explained. Hence, it is to difficult to establish the findings of the authors presumed to have captured.
6. The change of behavior among any person in any settings is dependent on many factors. For this study, the authors have relied on few variables like mother’s education and profession. However, there are many other important key factors like the type of housing, socio-economic status of the family, the type of school, the father’s education and profession, the siblings in their house, their exposure to social media etc., which were ignored in this study.
